# Incidence and mortality of alcohol‐related dementia and Wernicke‐Korsakoff syndrome: A nationwide register study

**DOI:** 10.1002/gps.5775

**Published:** 2022-07-05

**Authors:** Anniina Palm, Risto Vataja, Tiina Talaslahti, Milena Ginters, Hannu Kautiainen, Henrik Elonheimo, Jaana Suvisaari, Nina Lindberg, Hannu Koponen

**Affiliations:** ^1^ Department of Psychiatry University of Helsinki Helsinki Finland; ^2^ Helsinki University Hospital Helsinki Finland; ^3^ Primary Health Care Unit Kuopio University Hospital Helsinki Finland; ^4^ Folkhälsan Research Center Helsinki Finland; ^5^ Finnish Institute for Health and Welfare Helsinki Finland; ^6^ Mental Health Unit Helsinki Finland

**Keywords:** alcohol‐related dementia, incidence, mortality, Wernicke‐Korsakoff syndrome

## Abstract

**Background:**

Epidemiological data on alcohol‐related cognitive disorders are scarce. Up‐to‐date population‐based incidence and mortality rates for Wernicke‐Korsakoff syndrome (WKS) and alcohol‐related dementia (ARD) are necessary to understand the burden of these diseases.

**Methods:**

We collected diagnostic data from the Finnish Hospital Discharge Register and mortality data from Statistics Finland for all persons aged ≥40 years who had received a diagnosis of WKS (n = 1149) or ARD (n = 2432) between 1998 and 2015 in Finland. We calculated the incidences and mortality in relation to the age‐, sex‐ and calendar year‐matched general population. Causes of death were ascertained from death certificates.

**Results:**

For WKS, the incidence per 100,000 person‐years (95% confidence interval (CI)) was 3.7 (3.4–3.9) in men and 1.2 (1.1–1.3) in women. For ARD, the incidence was 8.2 (7.9–8.6) in men and 2.1 (1.9–2.3) in women. The incidence of WKS peaked in people aged 50–59 years and the incidence of ARD in people aged 70–79 years. The standardized mortality ratio (95% CI) was 5.67 (5.25–6.13) in WKS patients and 5.41 (5.14–5.70) in ARD patients. Most of the excess mortality resulted from alcohol‐related causes.

**Conclusions:**

To our knowledge, this is the first study describing population‐based incidence and mortality rates, sex‐segregated data and causes of death in patients with WKS or ARD. Our results establish a point of reference for the incidence of WKS and ARD and show the high mortality and poor prognosis of these disorders.

## Key points

- This study establishes previously unknown population‐based incidence and mortality rates for alcohol‐related dementia (ARD) and Wernicke‐Korsakoff syndrome (WKS).
- These are disorders with a poor prognosis, with patients over five times more likely to die in the follow‐up period than adults in the general population.
- The burden of premature mortality falls especially on younger (<65 years) age groups.
- The excess mortality of these disorders results from alcohol‐related causes of death.

## 1 BACKGROUND

Alcohol use disorders (AUDs) are common in most countries and cause significant morbidity and mortality. People with AUDs have a two‐to four‐fold greater risk of death (all‐cause mortality) than the general population, the risk being higher in younger age groups (≤40 years vs. ≥60 years) and higher in women than in men.^1^, ^2^, ^3^ Heavy drinking is associated with an elevated risk of all types of dementia.^4^

According to the current diagnostic systems, alcohol‐related cognitive impairment can be classified into two main syndromes: Wernicke‐Korsakoff syndrome (WKS) and alcohol‐related dementia (ARD).^5^ Wernicke encephalopathy (WE) is an acute neuropsychiatric disorder caused by thiamine (vitamin B1) deficiency. The clinical presentation is characterized by a classic triad of symptoms, namely mental status changes, oculomotor dysfunction and cerebellar dysfunction, although only a minority of patients present with all three. Up to 90% of WE cases in Western studies are related to alcohol misuse.^6^, ^7^, ^8^ Unrecognized or untreated, WE progresses to Korsakoff syndrome (KS) in around 56%–84% of alcoholic patients and possibly less frequently in cases unrelated to alcohol misuse. The principal cognitive sign of WKS is a profound amnesia, both anterograde and retrograde, although the disorder can cause a broader range of cognitive and behavioural symptoms.^7^, ^9^

The classification of ARD as a distinct clinical entity has been debated, mainly because of ambiguity concerning its aetiopathology, lack of defined diagnostic criteria and confounding factors in assessing the target population. Some authors have preferred the term ‘alcohol‐related brain damage’ to encompass neurocognitive disorders related to chronic alcohol use, including WKS and ARD.^5^ Uncertainty prevails as to whether ARD is a direct result of ethanol neurotoxicity (neurotoxicity hypothesis), represents another pathology (thiamine deficiency) or has a multifactorial aetiopathology.^5^, ^7^ Oslin et al.^10^ proposed provisional diagnostic criteria for ARD, suggesting that a 5‐year period of consuming on average 35 standard drinks per week for men (28 for women) is sufficient for developing the disorder. Diagnosis of ARD is often complicated by other conditions linked to abuse of alcohol such as head trauma, substance abuse, psychiatric comorbidities and a higher rate of vascular risk factors.^5^ While the neuropsychological profile of ARD has received limited attention, a more global cognitive decline than with WKS has been observed.^5^

Epidemiology of both WKS and ARD has been relatively seldom investigated, and reliable estimates of their prevalence and incidence are lacking. Much of the current knowledge of both disorders is derived from studies conducted in 1970–1990 and may be out of date. In older autopsy studies, the prevalence of WE was estimated to be 0.4%–2.8% in the general population^11^ and up to 12.5%–59% in alcoholics or in alcohol‐related deaths.^12^, ^13^, ^14^ Newer studies have also been limited in their scope or methodology. Two studies used health care admission data or questionnaires to estimate the prevalence of KS in the Netherlands to be 3–4.8 per 10,000 inhabitants.^15^, ^16^ In their retrospective study of hospital patients, Ramayya and Jauhar^17^ estimated the annual incidence of KS to increase from 12.5 to 81.25 per million inhabitants between 1990 and 1995 in Glasgow, Scotland, and cited six older studies that reported an annual incidence of 1–65 per million adults in 1961–1977. Up to 80% of WE cases confirmed post mortem were not diagnosed during the lifetime.^12^, ^18^

Prevalence estimates of ARD have also been notably variable and are often derived from population studies that correlate dementia rates and alcohol consumption or from studies investigating ARD as a proportion of dementias in some sub‐population. A review of nine epidemiological studies found that the prevalence of ARD ranged from 0.119% in multi‐day hospital admission patients to 25.6% in elderly alcoholics from substance abuse clinics.^19^ Population‐based studies have reported a prevalence of 0.0066% in those aged 30–64 years^20^ and 0.7% in US Medicare beneficiaries aged ≥68 years.^21^ A Spanish study reported an ARD incidence of 1.5 per 100,000 person‐years in men and 0.5 in women, based on a small number (n = 48) of ARD patients.^22^ Of early and late‐onset dementia cases, 1%–14%^23^ and 0.8–1.3%,^24^, ^25^ respectively, are attributed to ARD.

Data on the prognosis and long‐term mortality of WKS and ARD are scarce. An older case series of 245 WKS patients reported an acute mortality of 17%, death occurring on average 8 days after onset of symptoms, and an additional 26% of patients dying during follow‐up (average time to death 3.2 years). Mortality was mostly caused by infections, liver cirrhosis and carcinomas.^9^ Newer studies have described a 5.3%–10% acute mortality of WE patients.^26^, ^27^ Sanvisens et al.^26^ reported a median survival estimate of 8 years and a mortality rate of 7.4 per 100 person‐years in hospital patients diagnosed with alcohol‐related WE or KS. The main causes of death were bacterial infections and cancer.

There are few population‐based studies and, to our knowledge, no nationwide epidemiological studies on the incidence and mortality of WKS and ARD. Prevalence and incidence rates in earlier studies are extremely variable, owing to the heterogeneous study populations. Furthermore, almost no research exists on the long‐term mortality, natural progression and causes of death of these disorders. With this shortfall in mind, we decided to conduct this extensive nation‐wide register study. The objective of our study was to examine the current population‐based incidence of WKS and ARD, and mortality and causes of death of patients with these diagnoses.

## 2 METHODS

### 2.1 Register data: Diagnoses and mortality

Data on assessed patients were collected from the Finnish Hospital Discharge Register (FHDR, renamed as Care Register for Health Care).^28^ The FHDR includes inpatient care (register named Hoitoilmoitusjärjestelmä or Hilmo), outpatient visits in specialised health care (Hilmo) and primary care outpatient visits (Avohilmo). Inpatient data are collected from all public hospitals, municipal health centre wards and other (private, prison and military) hospitals, and contain main diagnoses and subdiagnoses of the hospital stay, information about procedures and interventions and basic patient characteristics such as age, sex and residence status. Information on outpatient visits in specialized health care is collected from public services only. The AvoHILMO contains information about all outpatient visits to public primary care since 2009. Data on population, mortality and causes of death were collected from Statistics Finland, which covers statistics on deaths of persons permanently domiciled in Finland. The study is based on register data only; individual clinical records or autopsy data were not accessed.

### 2.2 Study population

The study sample included all patients (n = 3581) who had received a diagnosis of WKS (n = 1149, 841 men and 308 women) or ARD (n = 2432, 1892 men and 540 women) between 1998 and 2015 in Finland, and who were aged ≥40 years at diagnosis. The lower age limit was chosen to scrutinize mortality during the follow‐up period, as mortality was expected to be higher in older age groups. The patients were followed from 1998 to 2015 or until death. Data on mortality from death certificates were collected until the end of 2018.

The classification of diagnoses was based on the 10th revision of the World Health Organization International Classification of Diseases (ICD‐10).^29^ The study population included all cases with ICD‐10 diagnoses F10.6 (alcohol‐induced amnesic syndrome) for WKS and F10.73 for ARD. In analysing causes of death, alcohol‐related diseases included all diseases caused by alcohol (ICD‐10 categories F10, G31.2, G40.51, G62.1, G72.1, I42.6, K29.2, K70, K85.2, K86.0, O35.4, P04.3, Q86.0).

### 2.3 Data analysis

The descriptive statistics were presented as means with standard deviations or as counts with percentages. Statistical comparisons between the groups were made by using the *t*‐test or Chi‐square test. Incidence rates (per 1000 person‐years) were calculated overall, and within strata defined by age and sex separately by diagnosis. Patients and the population at risk were stratified by gender, age and calendar (year) period. Incidence rates with 95% confidence intervals (CIs) were calculated assuming a Poisson distribution. Kaplan‐Meier's method was utilized to estimate cumulative proportions of survival, which were then compared between diagnoses with the log‐rank test. The Cox proportional hazard model was used to estimate the age‐adjusted risk for survival. Standardized mortality ratio (SMR), the ratio of observed to expected numbers of deaths, for all‐cause and alcohol‐related deaths was calculated using subject‐years methods with 95% CIs. The expected number was determined by multiplying the person‐years of observation by the appropriate mortality rate in the general population according to categories of gender, 1‐year age group and calendar (year) period. Statistical comparison between SMRs was made by using Poisson regression models. The Poisson regression models were evaluated using the goodness‐of‐fit test, and the assumptions of overdispersion in models were tested using the Lagrange multiplier test. Probabilities of survival and population at risk in an age‐ and gender‐matched sample of the general population were calculated from data of the Official Statistics of Finland from Statistics Finland. Stata 17.0 (StataCorp LP; College Station, TX, USA) statistical package was used for the analysis.

### 2.4 Ethical considerations

The study protocol was approved by the Ethics Committee of Helsinki University Central Hospital (186/13/03/00/16). Informed consent was not required due to the register‐based design.

## 3 RESULTS

### 3.1 Incidence and mortality

Table 1 shows the characteristics of the study population, incidences and mortality data organized by diagnosis and sex. Wernicke‐Korsakoff syndrome patients were significantly younger than ARD patients, the mean age at diagnosis being 57 years in the WKS group and 65 years in the ARD group; age at diagnosis was similar for men and women. Men represented 73.2% of WKS patients and 77.8% of ARD patients.

**TABLE 1:** Characteristics of the study population, incidences and mortality data by diagnosis and sex

Study population	WKS	ARD	*P*‐value
All			
Number	1149	2432	
Person‐years followed up	9191	12,620	
Number of deaths	645	1491	
Mean age at diagnosis, mean (SD)	57 (9)	65 (9)	<0.001
Incidence/100,000 pyrs (95% CI)	2.4 (2.2–2.5)	5.0 (4.8–5.2)	<0.001
Crude mortality^a^, % (95% CI)	79.0 (74.1–83.4)	91.6 (85.1–96.0)	<0.001
Standardized mortality ratio (95% CI)	5.67 (5.25–6.13)	5.41 (5.14–5.70)	0.32
<65 years	7.32 (6.70–8.01)	8.12 (7.51–8.78)	
65 years and over	3.46 (2.97–4.03)	4.37 (4.09–4.67)	
Median survival time, years (95% CI)	10.7 (9.6–11.3)	5.9 (5.6–6.3)	
Men			
Number	841	1892	
Person‐years followed up	6334	9584	
Number of deaths	511	1199	
Mean age at diagnosis, mean (SD)	57 (9)	65 (9)	<0.001
Incidence/100,000 pyrs (95% CI)	3.7 (3.4–3.9)	8.2 (7.9–8.6)	<0.001
Crude mortality*, % (95% CI)	85.2 (79.7–89.9)	92.2 (83.8–97.2)	<0.001
Standardized mortality ratio (95% CI)	5.58 (5.12–6.09)	5.16 (4.87–5.46)	0.13
<65 years	7.09 (6.41–7.84)	7.43 (6.80–8.11)	
65 years and over	3.48 (2.94–4.13)	4.24 (3.93–4.56)	
Median survival time, years (95% CI)	9.4 (8.3–10.5)	5.7 (5.3–6.0)	
Women			
Number	308	540	
Person‐years followed up	2857	3037	
Number of deaths	134	292	
Mean age at diagnosis, mean (SD)	57 (9)	65 (9)	<0.001
Incidence/100,000 pyrs (95% CI)	1.2 (1.1–1.3)	2.1 (1.9–2.3)	<0.001
Crude mortality^a^, % (95% CI)	60.7 (52.3–69.3)	92.4 (81.1–98.2)	<0.001
Standardized mortality ratio (95% CI)	6.05 (5.11–7.17)	6.81 (6.07–7.34)	0.26
<65 years	8.41 (6.91–10.24)	13.06 (10.94–15.58)	
65 years and over	3.37 (2.42–4.70)	5.05 (4.34–5.87)	
Median survival time, years (95% CI)	14.3 (11.6–18.0)	7.3 (6.4–8.7)	

^a^ At the end of the follow‐up period.

Abbreviations: ARD, alcohol‐related dementia; CI, confidence interval; pyrs, person‐years; SD, standard deviation; WKS, Wernicke‐Korsakoff syndrome.

**FIGURE 1:**
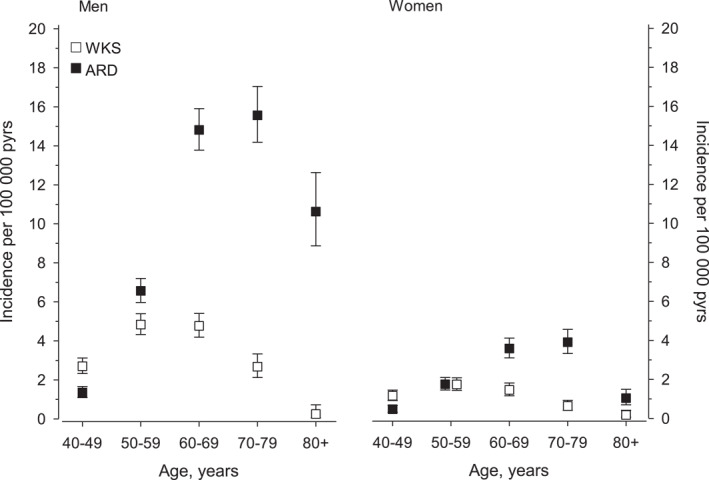
Incidence of Wernicke‐Korsakoff syndrome (WKS) and alcohol‐related dementia (ARD) in men and women per 100,000 person‐years. Abbreviations: ARD, alcohol‐related dementia; pyrs, person‐years; WKS, Wernicke‐Korsakoff syndrome

The incidence of WKS per 100,000 person‐years (95% CI) was 3.7 (3.4–3.9) in men and 1.2 (1.1–1.3) in women. The incidence of ARD was 8.2 (7.9–8.6) in men and 2.1 (1.9–2.3) in women. The peak incidence of WKS, in the age group of 50–59 years, was 4.8 (4.3–5.4) in men and 1.7 (1.4–2.1) in women. Alcohol‐related dementia was primarily diagnosed in older age groups, the peak incidence in the age group of 70–79 years being 15.6 (14.2–17.0) in men and 3.9 (3.3–4.6) in women (Figure 1).

The SMR (95% CI) was 5.67 (5.25–6.13) in the WKS group and 5.41 (5.14–5.70) in the ARD group. The SMRs were higher in younger persons: the SMR for people under 65 years was 7.32 (6.70–8.01) in the WKS group and 8.12 (7.51–8.78) in the ARD group. Women had a significantly higher SMR than men in the ARD group (*p* < 0.001), but not in the WKS group (*p* = 0.41) (Table 1).

**FIGURE 2:**
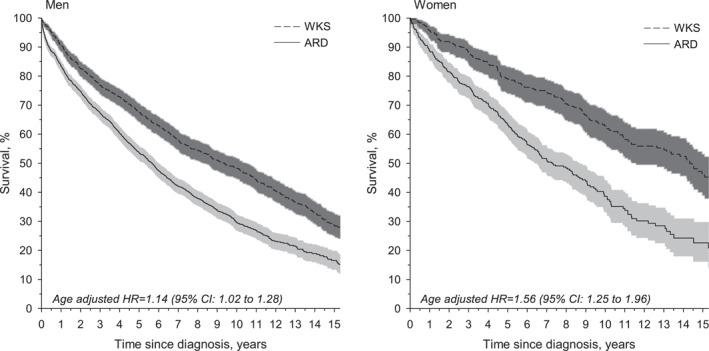
Survival in Wernicke‐Korsakoff syndrome (WKS) and alcohol‐related dementia (ARD) in men and women. Abbreviations: ARD, alcohol‐related dementia; CI, confidence interval (CI); HR, hazard ratio; pyrs, person‐years; WKS, Wernicke‐Korsakoff syndrome

Figure 2 shows the Kaplan‐Meier survival estimates for WKS and ARD. The 5‐year survival (95% CI) in the WKS group was 67.7% (64.3–70.7) for men and 79.0% (73.9–83.1) for women. The 10‐year survival (95% CI) was 48.3% (44.6–51.8) for men and 62.9% (56.7–68.3) for women. In the ARD group, the 5‐year survival was 53.4% (51.2–55.8) for men and 63.4% (59.0–67.4) for women, while the 10‐year survival was 29.5% (26.9–32.2) for men and 38.3% (27.0–44.0) for women.

### 3.2 Causes of death

The main causes of death for people with a diagnosis of WKS were diseases of the circulatory system (24.0%), neoplasms (16.4%), diseases of the digestive system (16.0%), mental and behavioural disorders (13.3%) and accidents, suicides and other external causes (12.1%). Of WKS patients, 32.6% died from alcohol‐related causes. Most of these deaths were caused by organ damage related to long‐term alcohol use such as alcoholic liver disease (13% of all deaths). About one‐third of alcohol‐related deaths resulted from mental and behavioural disorders due to alcohol use (ICD‐10 category F10) (Table 2).

**TABLE 2:** Causes of death

		WKS	ARD
Cause of death (ICD‐10 codes)		*N* = 645 n (%)	*N* = 1491 n (%)
A00‐B99	Infectious and parasitic diseases	4 (0.6)	4 (0.3)
C00‐D48	Neoplasms	106 (16.4)	225 (15.1)
E00‐E90	Endocrine, nutritional and metabolic diseases	13 (2.0)	17 (1.1)
F00‐F99	Mental and behavioural disorders	86 (13.3)	375 (25.2)
G00‐G99	Diseases of the nervous system	53 (8.2)	154 (10.3)
I00‐I99	Diseases of the circulatory system	155 (24.0)	372 (24.9)
J00‐J99	Diseases of the respiratory system	37 (5.7)	70 (4.7)
K00‐K93	Diseases of the digestive system	103 (16.0)	158 (10.6)
M00‐M99	Diseases of the musculoskeletal system and connective tissue	1 (0.2)	4 (0.3)
N00‐N99	Diseases of the genitourinary system	0 (0.0)	8 (0.5)
R00‐R99	Symptoms, signs and abnormal clinical and laboratory findings	8 (1.2)	5 (0.3)
V01‐Y98	External causes of morbidity and mortality	78 (12.1)	99 (6.6)
X45	Accidental poisoning by and exposure to alcohol	20 (3.1)	19 (1.3)
X60‐84	Intentional self‐harm	14 (2.2)	8 (0.5)
X85‐Y34	Assault	6 (0.9)	6 (0.4)
	Unknown	1 (0.2)	0 (0.0)
	Alcohol‐related diseases^a^	210 (32.6)	551 (37.0)

^a^ ICD‐10 categories F10, G31.2, G40.51, G62.1, G72.1, I42.6, K29.2, K70, K85.2, K86.0, O35.4, P04.3, Q86.0.

Abbreviations: ARD, alcohol‐related dementia; WKS, Wernicke‐Korsakoff syndrome.

In the ARD group, 37.0% of patients died from alcohol‐related causes; the majority (about 58%) of these deaths were caused by mental and behavioural disorders due to alcohol use (ICD‐10 category F10). The rest were mostly caused by other alcohol‐related organ damage such as liver and pancreatic diseases. Overall, the main causes of death of ARD patients were mental and behavioural disorders (25.2%), diseases of the circulatory system (24.9%), neoplasms (15.1%), diseases of the digestive system (10.6%), diseases of the nervous system (10.3%) and accidents, suicides and other external causes (6.6%) (Table 2).

In the WKS group, the SMR for alcohol‐related deaths (95% CI) was 24.12 (21.06–27.61). Stratified by sex, the SMR for men was 20.20 (17.27–23.63) and for women 54.77 (38.03–71.51). In the ARD group, the SMR for alcohol‐related deaths (95% CI) was 46.60 (42.87–50.66); the SMR for men was 39.30 (35.75–43.20) and for women 134 (112.42–160.32).

## 4 DISCUSSION

To our knowledge, this is the first study describing population‐based incidence and mortality rates, sex‐stratified data and causes of death of patients with WKS or ARD. As novel findings, our results establish up‐to‐date population‐based ARD and WKS incidence rates. We show that the burden of premature mortality of WKS and ARD falls especially on younger (<65 years) age groups. The excess mortality of these disorders results from alcohol‐related causes, as the SMRs for alcohol‐related deaths were notably high.

### 4.1 Incidence

Our nation‐wide study offers essential information about the clinical burden of WKS and ARD. The incidence of WKS in our study was 2.4 (95% CI: 2.2–2.5) per 100,000 person‐years in people aged ≥40 years. This result falls within the range reported in earlier studies, with KS incidence rates of 0.1–6.5/100,000 person‐years (in 1970–1990) and 1.25–8.125/100,000 person‐years (in the 1990s).^17^

Wernicke‐Korsakoff syndrome prevalence estimates from older post‐mortem studies have been consistently higher (around 0.4%–2.8%^11^) than those from clinical or register studies.^8^ It is possible that the prevalence of WKS has decreased from the 1960s to the 1980s, when most of the autopsy studies were performed, or that a part of the WE cases detected post‐mortem were caused by aetiology other than alcohol. However, these older studies reported that only a minority of WE patients were correctly diagnosed during the lifetime.^12^, ^18^ The clinically diagnosed WKS cases may be distorted towards those with a more severe clinical presentation.

In our study, the incidence of ARD was 5.0 (95% CI: 4.8–5.2) per 100,000 person‐years in people aged ≥40 years. As we found no earlier population‐based studies describing the incidence rates of ARD, our results establish a useful point of reference for population‐based ARD incidence. Like WKS, ARD may also often remain underdiagnosed.^30^ Our figures likely are conservative estimates of the incidence of WKS and ARD.

Both WKS and ARD affect younger age groups than many progressive neurocognitive disorders like Alzheimer's disease. Our findings indicate that WKS is a disorder of younger patients than ARD, with the highest WKS incidence rates in people aged 50–59 years, while ARD incidence peaked in the age group 70–79 years. These results are in line with earlier WKS studies, which have described a mean age at diagnosis or admission to long‐term care to be around 50–57 years^17^, ^31^ and a peak incidence age of 40–59 years.^9^, ^18^ Earlier ARD studies have reported the mean age of institutionalized or hospitalized ARD patients to be around 64–73 years.^30^, ^32^

Wernicke‐Korsakoff syndrome and ARD disproportionately affect men. About three‐quarters of people with either diagnosis were male in our study. This corresponds to earlier results, where men accounted for around 60%–75% of WKS patients^9^, ^17^, ^18^, ^31^ and 74–82% of ARD patients.^30^, ^32^ These numbers reflect the fact that most sufferers of AUDs are men; about 76%–77% of hospitalized Finnish AUD patients between 1997 and 2006 were male.^3^

### 4.2 Mortality

Our results demonstrate the high mortality and poor prognosis of these disorders. Patients with WKS and ARD were over five times more likely to die in the follow‐up period than adults in the general population. Women had a significantly higher SMR than men in the ARD group. There was no significant difference in SMRs between the sexes in the WKS group. The burden of premature mortality fell mostly on younger age groups, as the SMRs in our study were consistently higher in younger (<65 years) patients, especially women.

Most of the excess mortality in our study resulted from alcohol‐related causes. The SMRs for alcohol‐related deaths were notably high, 24.12 in the WKS group and 46.60 in the ARD group. Women had higher SMRs for alcohol‐related deaths than men in both diagnosis groups, reflecting that women are at greater risk for alcohol‐related morbidity and mortality, especially as they age.^2^

The causes behind excess mortality in ARD and WKS are likely multifactorial. Heavy drinking is known to affect the cardiovascular, gastrointestinal and immune systems. Heavy alcohol use is associated with liver diseases, pancreatitis, gastritis, various cancers and fatal accidents.^33^ Susceptibility to alcohol toxicity increases with age.^34^ In a Nordic register study, AUDs led to a 24‐ to 28‐year shorter life expectancy compared to the general population; the mortality rate ratio for Finnish people with AUDs was 3.0–3.2 in men and 3.5–4.0 in women.^3^ It is noteworthy that in our study WKS and ARD seem even more lethal, with an SMR of 5.4–5.7.

Our results show that survival rates of WKS and ARD patients drop steadily over a long follow‐up. This may be explained by frequent comorbidities and continuing the negative health behaviours that led to the disorder in the first place. In fact, Sanvisens et al.^26^ showed that two‐thirds of hospitalized WKS patients continued to use alcohol after discharge, and 6% presented with a new WE episode. Of KS patients in long‐term care, 68% had at least one somatic comorbidity, the most common ones being chronic obstructive pulmonary disease, cardiovascular disease, hypertension, diabetes mellitus, cerebrovascular accident, epilepsy and malignancy.^31^ KS patients were often unmarried, unemployed, living alone^17^ and smoked tobacco.^26^ ARD patients also commonly had medical comorbidities such as liver disease, infections, head injuries and epilepsy.^32^

### 4.3 Alcohol use and cognition

The amount of alcohol consumption necessary for developing WKS or ARD is uncertain. Acute WE results from about 2 to 6 weeks of thiamine deficiency.^6^, ^8^ Beside alcohol abuse, other factors, such as malnutrition and hepatic impairment, affect the level of thiamine depletion in alcoholics.^8^ Oslin et al.^10^ proposed that consuming 35 standard drinks a week (28 for women) for 5 years is sufficient to risk developing ARD. A binge‐withdrawal pattern of drinking may be especially harmful to cognition.^5^

The total annual alcohol consumption in Finland in 1998–2015 was 8.0–10.0 L of 100% alcohol per capita (or 9.8–12.1 L per inhabitant aged 15 years or over),^35^ which is nearly the same as the average European consumption.^36^ Drinking patterns are highly skewed; 10% of the population drinks about half of all the alcohol consumed in Finland, while another 10% are total abstainers.^37^

### 4.4 Strengths and limitations

This extensive nation‐wide register study included all patients from both inpatient and outpatient care with a documented alcohol‐induced WKS or ARD diagnosis in Finland between 1998 and 2015. The study had a large number of patients and a long follow‐up time. Quality and reliability of Finnish health care registers are considered to be high.^28^ We were able to ascertain the causes of death from death certificates, which are regarded as accurate.^38^

However, as a register‐based study, no individual clinical or autopsy data on the patients were available. The results are based on diagnoses made and documented during the standard course of care delivery, and the possibility of misdiagnosis cannot be excluded. Additionally, people abusing alcohol may not seek proper health care and may present with multiple confounding factors (such as comorbidities), which may further contribute to misdiagnosis. Our register data did not contain information on patients' risk factors, such as tobacco smoking or alcohol use. Our study included people aged 40 years and over only since WKS and ARD are rare in younger age groups.

### 4.5 Implications for clinical practice, research and policy

Our data indicate that WKS and ARD are significant public health problems that cause many years of lost life. Increasing awareness of the prevalence and the high morbidity and mortality of these disorders should improve diagnostics, prevention and care as well as promote research on these insufficiently studied disorders.

Wernicke‐Korsakoff syndrome and ARD are, in theory, fully preventable. General alcohol policies and selective efforts aimed at high‐risk alcohol abusers should be implemented with the goal of reducing alcohol‐related harm. Much of the morbidity and mortality associated with WKS might be prevented with early recognition and treatment of acute WE with parenteral thiamine according to established clinical guidelines.^39^ Screening for and treating somatic and psychiatric comorbidities in people with WKS and ARD should also be improved.

Unlike many other neurodegenerative disorders, ARD and WKS are non‐progressive and even partially reversible if abstinence can be achieved.^5^, ^40^ Our study shows that their excess mortality stems from alcohol‐related causes, suggesting that people with these disorders continue abusing alcohol and may not seek or receive necessary health care for alcoholism or comorbidities. Thus, even after the disorder has developed, efforts should be made to offer rehabilitation, care and support for alcohol abstinence.

## CONFLICT OF INTEREST

The author declares that there is no conflict of interest that could be perceived as prejudicing the impartiality of the research reported.

## Data Availability

Data may be obtained from a third party and are not publicly available. According to Finnish law, the data in the social welfare and health care registers and documents are confidential. Researchers who wish to replicate our study may apply for permission to use the registers of the Finnish Institute for Health and Welfare (info@thl.fi) for data from the Care Register for Health Care, and Statistics Finland (info@stat.fi) for data on population, mortality and causes of death.
